# Maren Pills Improve Constipation via Regulating AQP3 and NF-*κ*B Signaling Pathway in Slow Transit Constipation In Vitro and In Vivo

**DOI:** 10.1155/2020/9837384

**Published:** 2020-07-23

**Authors:** Yu Zhan, Xuegui Tang, Hong Xu, Shiyu Tang

**Affiliations:** ^1^Department of Anus and Intestine Surgery, Chengdu Integrated TCM &Western Medicine Hospital, Chengdu First People's Hospital, Chengdu, China; ^2^Department of Anus and Intestine Surgery, Affiliated Hospital of North Sichuan Medical College, Nanchong, China

## Abstract

**Background:**

Maren pills have been used to treat constipation. Aquaporin 3 (AQP3) plays a vital role in regulating water transfer in the colon. It has been reported that the downregulation of AQP3 can regulate liquid water metabolism and intestinal permeability in irritable bowel syndrome (IBS) rats' colon via NF-*κ*B pathway. In this study, we investigated whether the laxative effect of Maren pills is associated with the regulation of AQP3 and NF-*κ*B signaling pathway in the colon.

**Methods:**

The compound diphenoxylate suspension-induced STC rats received Maren pills intragastrically for 1 consecutive week to evaluate the laxative effect of Maren pills involving the regulation of AQP3 and NF-*κ*B signaling pathway. Moreover, human intestinal epithelial cells (HT-29) were treated with drug serum to obtain in vitro data.

**Results:**

Our results revealed that treatment with Maren pills increased the stool number, moisture content of feces, and intestinal transit rate in a dose-dependent manner. Maren pills significantly increased the AQP3, fibrosis transmembrane conductance regulator (CFTR), and protein kinase A (PKA) proteins in the colon of rats and in HT-29 cells. Mechanistically, Maren pills obviously inhibited the activation of NF-*κ*B pathway in the colon of rats and in HT-29 cells.

**Conclusion:**

These results suggest that the laxative effect of Maren pills is associated with the increased expression of AQP3 by downregulating NF-*κ*B signal pathway.

## 1. Introduction

Slow transit constipation (STC) is a common type of functional constipation, characterized by difficulty, infrequent, or incomplete defecation [1]. Epidemiological studies show that STC has a high incidence in the world, up to 3.19–11.6% in China, and has become one of the important factors affecting people's quality of life [2, 3]. At present, regulation of the gastrointestinal tract is the primary focus of constipation therapies. Mosapride is a novel serotonin type-4 (5-HT4) receptor agonist, which can enhance gastrointestinal motility and is widely used in the clinical treatment of constipation [4]. However, serious side effects can be observed as well as failure of therapy. In addition, high variable success rate (39–100%) for surgical treatment of STC has been reported [5]. Therefore, it is urgent to find adjuvant therapy for STC.

In recent years, a variety of herbal medicines and traditional Chinese formula have drawn attention as a new treatment for STC. Maren pills are a Kampo medicinal formula composed of six medicinal herbs, includes Rhubarb, *Magnolia officinalis*, Fructus aurantii immaturus, Semen cannabis, Apricot kernel, and *Paeonia lactiflora* Pall [6]. In clinical practice, Maren pills have been proved to increase the defecating frequency and improve clinical symptoms of patients with constipation [7]. However, the molecular mechanism involved in the laxative effect of Maren pills has not been elucidated.

Aquaporins (AQPs) are water and water/glycerin channels responsible for the rapid transport of water through the membrane and are expressed in various organs [8]. Currently, 13 types of AQPs have been identified in mammals, including AQP0-AQP12. Among them, AQP3 is the most important aquaporin in the colon, which is closely related to chronic constipation [9]. Many studies have demonstrated that AQP3 mediated the cathartic effect of laxatives [8, 10]. The nuclear factor-kappa B (NF-*κ*B) plays an important role in the process of immune response and inflammatory response. It has been reported that the AQP3 regulates liquid water metabolic abnormalities and intestine permeability alteration in irritable bowel syndrome (IBS) rats via NF-*κ*B pathway [11]. Another research reported that AQP3-facilitated H2O2 transport is required for NF-*κ*B activation in keratinocytes in the development of psoriasis [12]. Since water transfer is primarily mediated by AQP3, and mayuan tongbian zhitong decoction has been reported to downregulate the expression of AQP3 in STC rats, we hypothesized that the laxative effect of Maren pills may be associated with the regulation of AQP3 in the colon mediated by NF-*κ*B activation. Our research was to investigate the effect of Maren pills on AQP3 protein and NF-*κ*B signals in the STC rat models, and the results were further confirmed in HT-29 cells.

## 2. Materials and Methods

### 2.1. Animals and Treatment

Thirty healthy SD rats (230 ± 20 g, three months old) were purchased from the Animal Center of the West China Medical College of Sichuan University, Chengdu, China. All animals were kept in the animal laboratory under a temperature-controlled environment (23 ± 2°C), 55% ± 5% relative humidity, and 12 h light-dark cycle. They were allowed free access to food and water during the experimental session. After 1 week of adaptive feeding, the rats were randomly divided into 5 groups, with 6 rats in each group, including control, model, Maren pills (1 g/kg), Maren pills (0.5 g/kg), and Maren pills (0.25 g/kg) groups. The rats in the model and Maren pills groups received 10 ml/kg compound diphenoxylate suspension by intragastric administration, and the treatment was given once a day for 14 consecutive days to establish a constipation model. Therapeutic intervention was initiated the next day after the constipation model. The rats in the Maren pills groups received 0.25, 0.5, and 1 g/kg Maren pills intragastrically, and the treatment was given once a day for 1 consecutive week. The rats in the control and model groups received an equal volume of saline by intragastric administration. The body weight was determined using an automatic electronic balance after the last treatment. The feces of rats were collected 6 hours after the last treatment. Rats were sacrificed by cervical dislocation after anesthesia by intraperitoneal injection of 3% pentobarbital sodium (50 mg pentobarbital/kg rat body weight). The colon tissues were rapidly dissected, one part colon was flash-frozen in liquid nitrogen and then stored at−80°C, and another part was fixed in 4% paraformaldehyde until further study. The experimental protocol for the care and use of laboratory animals was approved by the Experimental Animal Ethics Committee of the West China Hospital of Sichuan University (Chengdu, China).

### 2.2. Measurement of Intestinal Transit Rate

After the treatment administration, all rats were fasted for 24 h but were allowed to drink freely. Subsequently, each rat was administered 1 ml of charcoal meal (3% suspension of activated charcoal in 0.5% aqueous methylcellulose; Sigma-Aldrich) by oral gavage. 30 min later, rats were sacrificed by cervical dislocation after anesthesia by intravenous injection of 1% pentobarbital (50 mg pentobarbital/kg rat body weight). Rats were put on a surgery platform. Then the abdomen was opened, the small intestine (the cecum front end) was taken out from the pylorus to ileocecal area and laid flat on a clean glass plate without traction, the full length and the distance from the front end of carbon powder to the pylorus were measured, and the ratio to the full length, namely, the intestinal transit rate = the propulsion distance of charcoal meal within the intestine (cm)/full length of the small intestine (cm) × 100%, was calculated.

### 2.3. Fecal Water Content

Detailed procedures have been previously described [1]. Briefly, fecal samples were placed in silica gel (Yubao, Shandong, China) followed by drying for 24 h in a desiccator. The fecal water content per gram of feces was calculated based on the difference between the wet and dry fecal weights. The percentage of water in the feces was calculated as follows: (wet weight − dry weight)/wet weight × 100%.

### 2.4. Cell Culture

Human colon cancer HT-29 cells were purchased from the Type Culture Collection of the Chinese Academy of Sciences, Shanghai, China. HT-29 cells were cultured in sterile Dulbecco's modified Eagle's medium (Hyclone, UT, USA) supplemented with 10% FBS and 1% penicillin-streptomycin under a humidified incubator with 5% CO_2_ at 37°C.

### 2.5. Preparation of Serum Containing Drugs

Twenty healthy SD rats, three months old and with a body weight of 230 ± 20 g, were purchased from the Animal Center of the West China Medical College of Sichuan University, Chengdu, China. After 1 week of adaptive feeding, the rats were randomly divided into 2 groups, with 10 rats in each group: the A group (received normal saline) and the B group (received 1 g/kg Maren pills). The blood of the rat abdominal artery was collected 6 h after the treatment. The serum was separated, sterilized by filtration, and stored at−80°C.

HT-29 cells (6 × 10^4^ cells/ml) were seeded in 6-well plates and allowed to reach 70–80% confluence and then intervened with drug-containing serum for 24 h. The cells were randomly divided into 5 groups, with 6 repeats, the control I (blank control), the control II (serum A), the Maren pills I group (serum B), the Maren pills II group (serum B + PMA), and PMA (phorbol-12-myristate-13-acetate).

### 2.6. HE Staining

The colon of the rat was fixed in 4% paraformaldehyde for 24 h. The colon tissues were dehydrated with ethanol and xylene, fixed in paraffin, and sectioned (4 *μ*m) for staining. The colon sections were stained with hematoxylin and eosin (H&E), and the images were acquired under a light microscope equipped with 10× or 40× objective lens.

### 2.7. Measurement of Inflammatory Factors

The colon was homogenized with PBS at a ratio of 1 : 9 (weight : volume) with a glass homogenizer in ice and centrifuged at 3500 rpm at 4°C for 10 min to obtain supernatant. HT-29 cells from each group were collected and digested with trypsin. Then, the cells were disrupted at 4°C by an ultrasonic cell disruptor and the lysate was centrifuged at 1000°r/min at 4°C for 10 min to obtain 100 *μ*l supernatant. Interleukin-1*β* (IL-1*β*), tumor necrosis factor-*α* (TNF-*α*), cyclooxygenase-2 (COX-2), motilin (MTL), and gastrin (GAS) concentration in colon supernatant from all experiment groups were detected by a commercial ELISA kit (IL-1*β*: H002; TNF-*α*: H052; COX-2: H200; MTL: H182; GAS: H239; Nanjing Jiancheng Bioengineering Institute, Jiangsu, China) according to the instructions strictly. Finally, the absorbance was measured at 450 nm on a microplate reader (Thermo Fisher Scientific, USA).

### 2.8. Western Blot Analysis

Total protein was extracted from colon tissue and HT-29 cells using ice-cold RIPA lysis buffer (Wuhan Boster Biological Technology, Ltd.), and the protein concentrations were measured using a BCA protein assay kit (Boster). Equal amounts (20 *µ*g) of protein was separated with 10% SDS-PAGE gel, then transferred onto PVDF membranes (EMD Millipore), and then blocked with 5% skim milk in TBST for 1 h at room temperature, and the membranes were incubated at 4°C overnight with primary antibodies against IKB*α* (ab7217; 1 : 2,000), p-p65 (ab86299; 1 : 5,000), p-IKK-*β* (ab59195), AQP3 (ab125219; 1 : 2,000), CFTR (ab2916; 1 : 2,000), PKA (ab38949; 1 : 2,000), and *β*-actin (ab8227; 1 : 1,000) all from Abcam (CA, USA). Then, the membranes were incubated with HRP-conjugated goat anti-rabbit immunoglobulin G (ab2057184; 1 : 5,000) for 1 h at room temperature. Proteins were visualized with the ECL western blotting detection reagents (Millipore). *β*-Actin was used as a loading control.

### 2.9. Immunofluorescence Assay

The colon of the rat was fixed in 4% paraformaldehyde for 24 h and embedded in paraffin, and tissue sections (5 *μ*m) of the colon were prepared for immunofluorescence analysis. For in vitro experiments, HT-29 cells were plated in 12-well plates and treated with drug serum or PMA (1 *μ*M; Beyotime, Shanghai, China) for 24 h. After washing with PBS twice, cells were fixed in 4% paraformaldehyde for 10 min at room temperature and prepared for immunofluorescence analysis.

For immunofluorescence analysis, tissue sections (5 *μ*m) of the colon or HT-29 cells were permeabilized with 0.1% Triton X-100, blocked with 5% BSA, and then incubated with primary rabbit anti-AQP3 antibody (ab125219; Abcam, CA, USA) overnight at 4°C. Then, the tissue and cells were washed twice with PBS/0.1% Tween-20 and incubated with a secondary goat anti-rabbit antibody conjugated with Alexa Fluor 488 for 1 h at room temperature. Then, the tissue and cells were reacted with 4, 6-diamidino-2-phenylindole (DAPI) solution (Beyotime) in PBS at room temperature for 5 min and then observed under a fluorescence microscope (Olympus Corporation, Tokyo, Japan) and photographed. The expression level of AQP3 in rat colon or HT-29 cells was analyzed by ImageJ software.

### 2.10. Statistical Analysis

Statistical analysis was performed using SPSS20.0 software (IBM Corp., Armonk, NY, USA). Values were presented as the means ± standard deviation (SD). Differences among multiple groups were compared by one-way analysis of variance (ANOVA) with Tukey's post hoc test. The differences were considered statistically significant at *P* < 0.05 and *P* < 0.01.

## 3. Results

### 3.1. Maren Pills Induce Diarrhea in Rats

**Figure 1:**
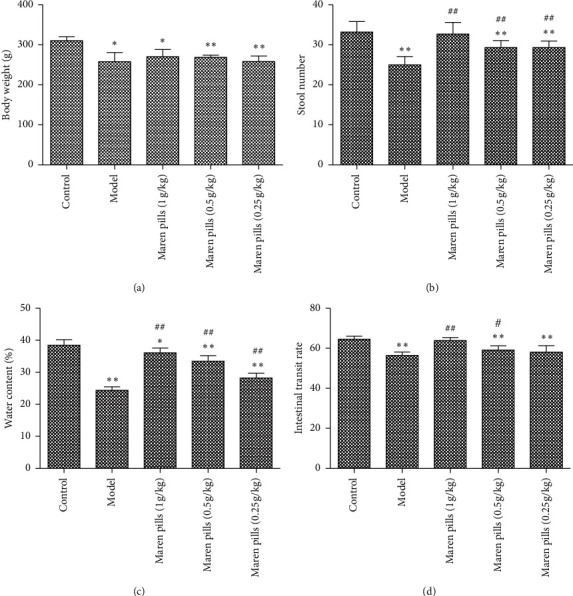
Effect of Maren pills on stool number and moisture content in diphenoxylate-induced STC rats. (a) No obvious change in body weight in experimental groups. (b) Maren pills dose-dependently (1, 0.5, and 0.25 g/kg) increased the stool number compared with the model group. (c) Maren pills dose-dependently (1, 0.5, and 0.25 g/kg) increased the fecal water content compared with the model group. (d) Maren pills dose-dependently (1, 0.5, and 0.25 g/kg) increased the intestinal transit rate compared with the model group. The results were presented as the mean ± standard deviation. ^*∗*^*P* < 0.05 and ^*∗∗*^*P* < 0.01, compared with the control group. ^#^*P* < 0.05 and ^##^*P* < 0.01, compared with the model group.

Rats did not die in the whole experiment, indicating that the STC model has good safety and operability. Compared with the control group, the rats in the model group presented fleeciness, dry stool, and decreased activity 4 days after modeling, and the symptoms were relieved after Maren pills treatment (data not shown). As shown in Figure 1, there was a significant weight loss in the other groups compared to the control group. In addition, rats with STC showed the stool number and moisture content of feces were significantly decreased, while the treatment of Maren pills improved the differences (Figures 1(b) and 1(c)). Taken together, these data indicated that the systemic administration of Maren pills did not show any alteration of body weight and had an effect on fecal water content.

### 3.2. Maren Pills Increase the Intestinal Transit Rate in Diphenoxylate-Induced STC Rats

As shown in Figure 1(d), the intestinal transit rate in the model group was significantly lower than that in the control group. However, the intestinal transit rate in 1 g/kg Maren pills- and 0.5 g/kg Maren pills-treated groups was significantly higher than that in the model group. These results indicated that Maren pills can improve the intestinal transit rate in diphenoxylate-induced STC rats.

### 3.3. Maren Pills Enhance the Colonic Motility Index Function and Alleviate the Inflammatory Response in Diphenoxylate-Induced STC Rats

**Figure 2:**
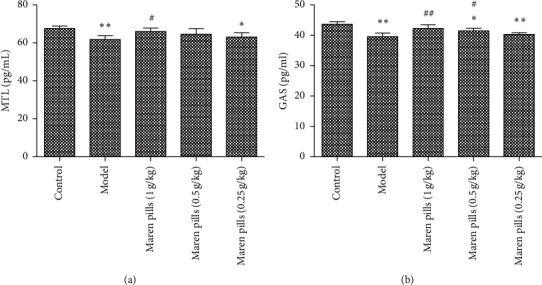
Effect of Maren pills on the colonic motility index function in diphenoxylate-induced STC rats. (a) Maren pills dose-dependently (1, 0.5, and 0.25 g/kg) increased the serum level of MTL compared with the model group. (b) Maren pills dose-dependently (1, 0.5, and 0.25 g/kg) increased the serum level of GAS compared with the model group. The results were presented as the mean ± standard deviation. ^*∗*^*P* < 0.05 and ^*∗∗*^*P* < 0.01, compared with the control group. ^#^*P* < 0.05 and ^##^*P* < 0.01, compared with the model group.

**Figure 3:**
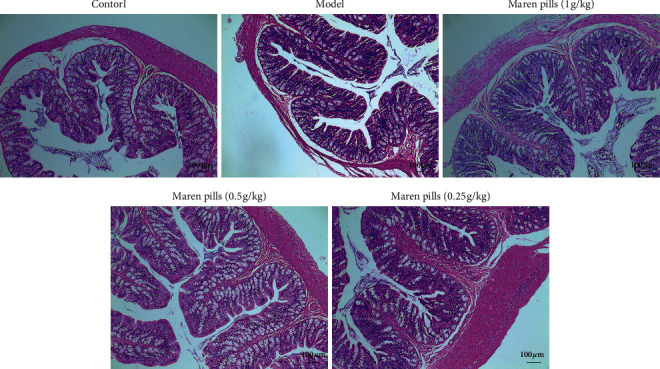
Histological morphology of colon with H&E staining after Maren pills treatment (scale bar, 100 *µ*m).

**Figure 4:**
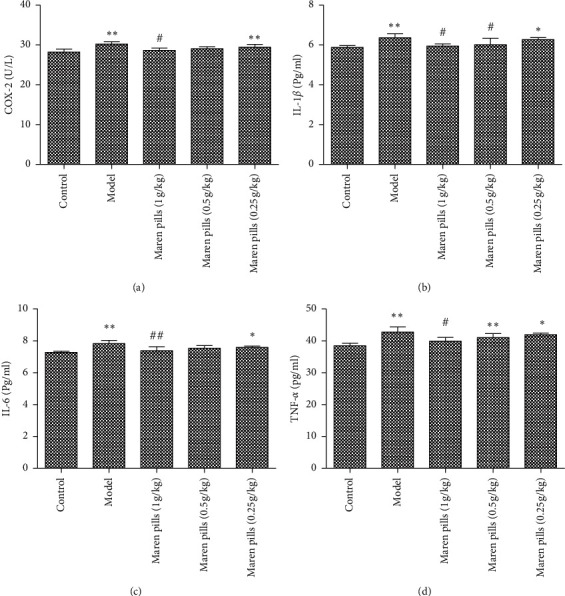
Effect of Maren pills on inflammatory factor in diphenoxylate-induced STC rats. Maren pills dose-dependently (1, 0.5, and 0.25 g/kg) decreased the contents of (a) COX-2, (b) IL-1*β*, (c) IL-6, and (d) TNF-*α*. The results were presented as the mean ± standard deviation. ^*∗*^*P* < 0.05 and ^*∗∗*^*P* < 0.01, compared with the control group. ^#^*P* < 0.05, compared with the model group.

**Figure 5:**
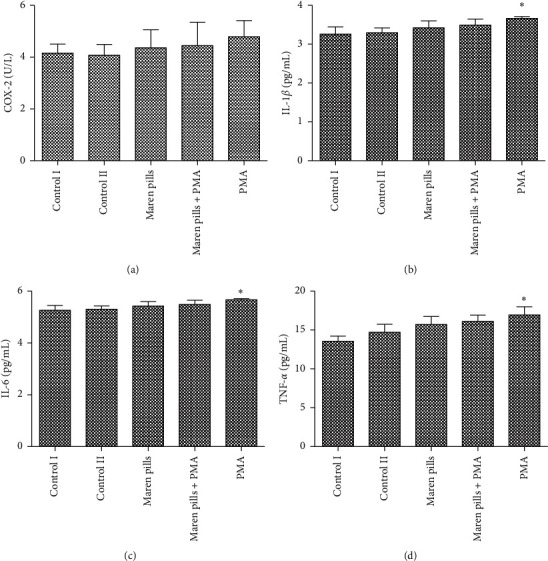
Effect of Maren pills on inflammatory factor in HT-29 cells. HT-29 cells were incubated with Maren pills or combined with PMA for 24 h. (a) COX-2 content in each group. (b) IL-1*β* content in each group. (c) IL-6 content in each group. (d) TNF-*α* content in each group. The results were presented as the mean ± standard deviation. ^*∗*^*P* < 0.05, compared with the control II group.

As shown in Figure 2, the serum levels of GAS and MTL were significantly decreased in the model group compared with the control group. However, the administration of Maren pills can effectively upregulate the serum levels of GAS and MTL compared with the model group. These data indicated that the intervention with Maren pills enhanced the colonic motility index function in STC rats. Infiltration of inflammatory cells into the mucosa was observed in the STC rats. However, Maren pills treatment decreased the infiltration of inflammatory cells (Figure 3). The ELISA results also showed that Maren pills decreased the contents of COX-2, IL-1*β*, IL-6, and TNF-*α* (Figure 4). Although there were statistical differences between the groups, the differences were very small, indicating that the above four proinflammatory factors may not be the action point of Maren pills. The action point of Maren pills remains to be further studied. In this study, the content of COX-2, IL-1*β*, IL-6, and TNF-*α* was also investigated in HT-29 cells. As shown in Figure 5, there was no significant effect on the content of inflammatory cytokines in the HT-29 cells treated with Maren pills. This may be because the HT-29 cells themselves have no inflammation.

### 3.4. Maren Pills Increase the Expression of AQP3 in STC Rats and HT-29 Cells

**Figure 6:**
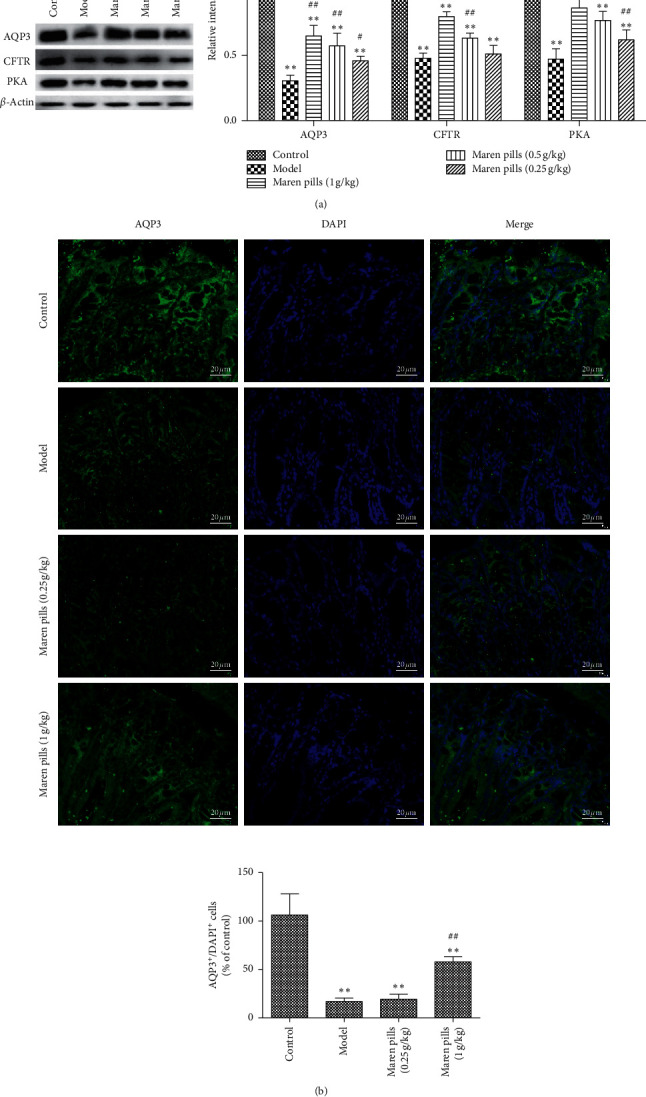
Effect of Maren pills on the expression of AQP3, CFTR, and PKA proteins in the colon of rats. (a) Maren pills dose-dependently (1, 0.5, and 0.25 g/kg) increased the expression of AQP3, CFTR, and PKA proteins compared with the model group. (b) The localization of AQP3 (green) in both the apical and lateral mucosal epithelial cells in the colons of STC rats and normal rats was determined by immunofluorescence staining. Cell nuclei (blue) were stained with DAPI (scale bar, 20 *µ*m). The results were presented as the mean ± standard deviation. ^*∗*^*P* < 0.05 and ^*∗∗*^*P* < 0.01, compared with the control group. ^#^*P* < 0.05, compared with the model group.

As shown in Figure 6(a), the expression levels of AQP3, CFTR, and PKA proteins in the model group were significantly decreased compared with the control group. Compared with the model group, Maren pills were markedly increased the expression levels of AQP3, CFTR, and PKA proteins in a dose-dependent manner. Further, the immunofluorescence was applied to confirm the location of AQP3 in colon tissues. The results revealed that AQP3 was present in both the apical and the lateral colonocytes of rats, and the treatment of Maren pills can significantly increase the fluorescence signal in colonocytes compared with the model group (Figure 6(b)). These results suggested that Maren pills increased the expression of AQP3 in the colon of rats.

**Figure 7:**
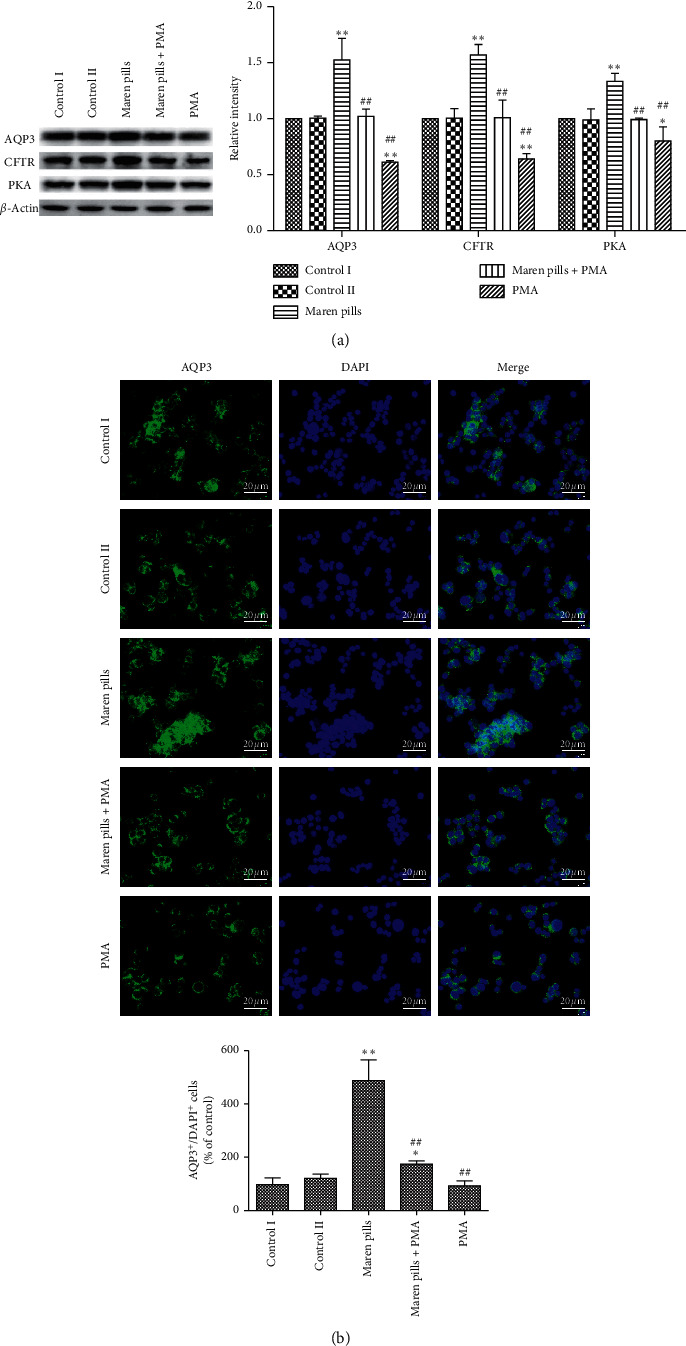
Effect of Maren pills on the expression of AQP3, CFTR, and PKA proteins in HT-29 cells. HT-29 cells were incubated with Maren pills or combined with PMA for 24 h. (a) The expressions of AQP3, CFTR, and PKA proteins in HT-29 cells were detected by western blot. (b) The localization of AQP3 (green) in HT-29 cells was determined by immunofluorescence staining. Nuclei were stained with DAPI (blue) (scale bar, 20 *µ*m). The results were presented as the mean ± standard deviation. ^*∗*^*P* < 0.05 and ^*∗∗*^*P* < 0.01, compared with the control II group. ^##^*P* < 0.01, compared with the Maren pills group.

In this study, the expressions of AQP3, CFTR, and PKA were also investigated in HT-29 cells. As shown in Figure 7(a), the AQP3, CFTR, and PKA protein expression levels increased significantly after Maren pills treatment. Similar to western blot analysis, immunofluorescence assay further confirmed that Maren pills increased the expression of AQP3, and AQP3 was expressed in cytoplasm and nucleus (Figure 7(b)). To clarify the relationship between AQP3 and NF-*κ*B, PMA (NF-*κ*B activator) was applied. The increased expression of AQP3 induced by Maren pills was significantly inhibited by PMA (Figures 7(a) and 7(b)).

### 3.5. Maren Pills Inhibit the Activation of NF-*κ*B Pathway in STC Rats

**Figure 8:**
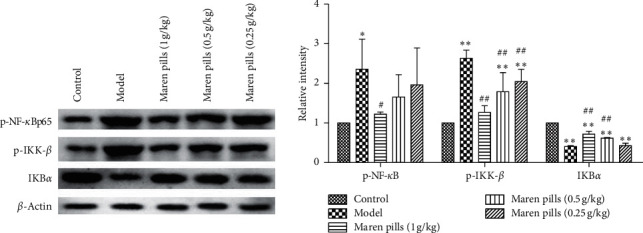
Effect of Maren pills on NF-*κ*B pathway in the colon tissue of STC rats. Maren pills dose-dependently (1, 0.5, and 0.25 g/kg) decreased the phosphorylation of NF-*κ*B and IKK-*β* and increased the expression of IKB*α* compared with the model group. The results were presented as the mean ± standard deviation. ^*∗*^*P* < 0.05 and ^*∗∗*^*P* < 0.01, compared with the control group. ^#^*P* < 0.05, compared with the model group.

The NF-*κ*B pathway has been reported to be involved in various types of diarrhea, and activation of the NF-*κ*B pathway is critical to the AQP3 channel [13]. As shown in Figure 8, Maren pills significantly decreased phosphorylation of NF-*κ*B and IKK-*β*, while significantly induced the expression of IKB*α*, indicating that Maren pills can inhibit the activation of NF-*κ*B signals in STC rats.

**Figure 9:**
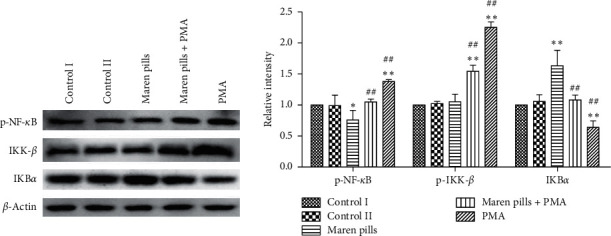
Effect of Maren pills on NF-*κ*B pathway in HT-29 cells. HT-29 cells were incubated with Maren pills or combined with PMA for 24 h. The expression levels of p-NF-*κ*B, p-IKK-*β*, and IKB*α* proteins in HT-29 cells were detected by western blot. The results were presented as the mean ± standard deviation. ^*∗*^*P* < 0.05 and ^*∗∗*^*P* < 0.01, compared with the control II group. ^##^*P* < 0.01, compared with the Maren pills group.

In this study, the NF-*κ*B pathway was also investigated in HT-29 cells. As shown in Figure 9, the p-NF-*κ*Bp65 protein expression level decreased significantly after Maren pills treatment, while IKB*α* expression was significantly increased. Moreover, the treatment of PMA (NF-*κ*B activator) significantly decreased the expression of p-NF-*κ*B and p-IKK-*β* proteins, while increased the expression of IKB*α*. These data indicated that Maren pills inhibited the activation of NF-*κ*B signals in HT-29 cells.

## 4. Discussion

STC is a common functional constipation characterized by slow colonic motility and delayed discharge of intestinal contents [14]. Maren pills have been used to treat constipation. However, the mode of action and potential drug targets of Maren pills in treating constipation are not clear. In the present study, to elucidate the mechanism of the laxative effect of Maren pills, we here discovered and demonstrated the effect of Maren pills on AQP3 and NF-*κ*B pathway both in vivo and in vitro. The main findings of our study are as follows: (1) Maren pills have the laxative effect via increasing intestinal fluid accumulation and intestinal motility, (2) Maren pills reduce inflammatory cell infiltration and increase muscle thickness, (3) Maren pills upregulate the AQP3 protein expression in the colon of rats and HT-29 cells, and (4) Maren pills inhibit the activation of NF-*κ*B signaling pathway in the colon of rats and HT-29 cells.

The pathogenesis of STC involves various mechanisms, and the compound difenolone suspension-induced STC is the most common method for studying functional constipation in animal models [15]. The rats treated with compound difenolone suspension displayed reduced intestinal peristalsis and fecal water content, which was similar to the clinical symptoms and pathological development of STC in humans. In order to verify the efficacy of Maren pills in the treatment of STC, we detected the effects of Maren pills in compound difenolone suspension-induced STC rat via evaluating stool number, fecal water content, intestinal transport rate, and histological procedure. Following the administration of Maren pills, obvious watery stool in the colon, increased stool number, fecal water content, and intestinal transport rate and decreased infiltration of inflammatory cells in the colon were observed, which is consistent with the data of the previous study [16]. These data indicated that Maren pills have the laxative activity partly via increasing intestinal fluid accumulation and intestinal motility.

AQP1-4 and AQP8 are known to be expressed in the mucosal epithelial cells of the colon in animals [17]. Current studies have shown that the pathogenesis of STC is closely related to the abnormality of colonic aquaporin [18]. Chao and Zhang [11] confirmed that the abnormal expression of AQPs can affect the changes in colonic water metabolism and intestinal permeability. In the present study, it revealed that Maren pills significantly increased the expression of AQP3 protein in both apical and lateral mucosal epithelial cells in the colon. In addition, a correlation was observed between this increase in the AQP3 protein expression and increase in fecal water content and intestinal transit rate. These results indicate that Maren pills can upregulate the AQP3 protein level in the epithelial cells of the colon mucosa, which prevent the reabsorption of water in the lumen by blood vessels, thus leading to diarrhea and improving constipation. The phosphorylation of CREB is mediated by protein kinases such as PKA, MAPK, PKC, and CaMKs, and phosphorylation of CREB can increase the transcription and expression of AQP3 [19]. Zhou et al. [20] revealed that vasoactive intestinal peptide improves constipation via regulating VIP-cAMP-PKA-AQP3 signaling pathway in STC rats. Activation of CFTR chloride channel is the main pathway to drive intestinal fluid secretion and plays an important role in maintaining the lubrication of luminal contents [21]. Harada et al. [22] demonstrated that Mashiningan improved opioid-induced constipation via activating CFTR chloride channel. In this study, we also found that Maren pills can upregulate the expression of PKA and CFTR proteins, which is consistent with the upregulated trend of AQP3, and jointly plays a regulatory role in constipation.

In addition to these findings, we further explored the precise mechanism involved in the increase of AQP3 of Maren pills, since inhibiting NF-*κ*B pathway is involved in promoting AQP3 expression [12, 23]. There was a study found that the activation of NF-*κ*B signals is important for the downregulation of AQP2 channel [17]. Hasler et al. [24] found that once NF-*κ*B was activated by hypertonic medium, it could reduce the expression of AQP2 by binding to the AQP2 promoter. In the study, we found that Maren pills markedly decreased phosphorylation of NF-*κ*B and IKK-*β* in the colon of rats, as opposed to an increase observed in the AQP3 expression level. The increased expression of AQP3 induced by Maren pills was effectively inhibited by PMA (NF-*κ*B activator). These results suggest that the increasing effect of Maren pills on AQP3 is partly by the downregulation of NF-*κ*B signal pathway.

## 5. Conclusion

In conclusion, our study showed that Maren pills can significantly improve the amount of defecation, fecal water content, and intestinal transport rate of STC rats and the mechanism may be related to the increased expression of AQP3 by downregulating NF-*κ*B signal pathway. This study revealed the reliable scientific basis for the treatment of STC by Maren pills, laying a foundation for further exploration of other relevant mechanisms.

## Data Availability

The datasets used or analyzed during the current study are available from the corresponding author on reasonable request.
